# Correction: Atlastins remodel the endoplasmic reticulum for selective autophagy

**DOI:** 10.1083/JCB.20180418510152018c

**Published:** 2018-11-05

**Authors:** Jin Rui Liang, Emily Lingeman, Saba Ahmed, Jacob E. Corn

Vol. 217, No. 10, October 1, 2018. 10.1083/jcb.201804185.

The immunofluorescence image in Figure 2 I was mislabeled as GFP-LC3B but is in fact immunofluorescence staining of endogenous LC3B. This information was correctly described in the original main text and figure legend and therefore does not affect the conclusion of the report.

**Figure 2.**
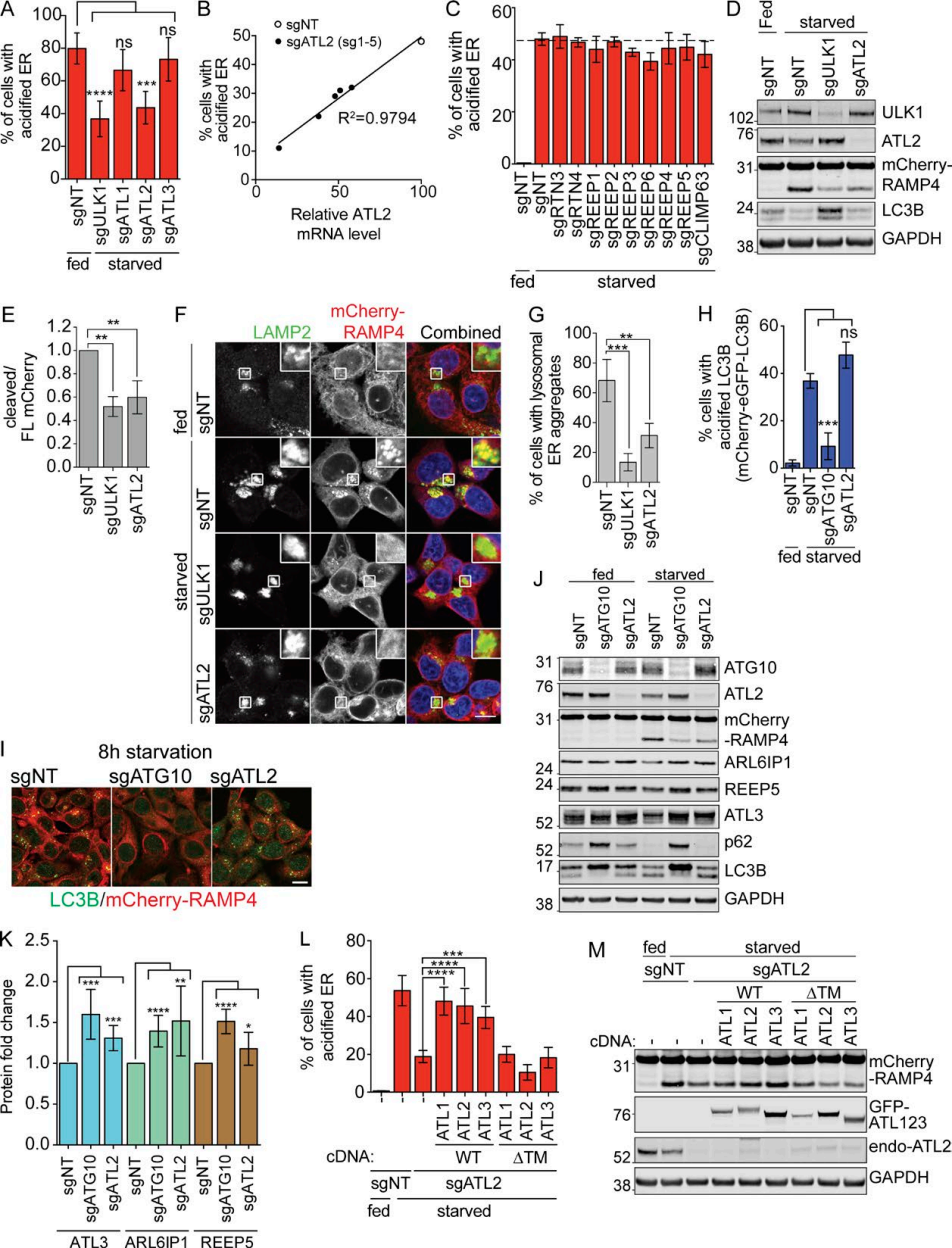
**EATR and CCER detect changes in ER-phagy induced by manipulation of a known ER-phagy receptor. (A)** EATR CRI​SPRi HCT116 cells expressing the indicated proteins were starved before FACS measurement. Data presented as mean ± SD of three biological replicates. P value indicates one-way ANO​VA with Dunnett’s multiple comparisons test (***, P < 0.001; ****, P < 0.0001). **(B)** The extent of ER-phagy inhibition of five different sgRNAs targeting ATL2 correlates with the knockdown efficiency of each sgRNA as determined by qRT-PCR. **(C)** EATR CRI​SPRi HCT116 cells transduced with the indicated sgRNAs were starved for 16 h before FACS measurement. Data presented as mean ± SD of three biological replicates. No statistically significant difference is determined between sgNT and the other sgRNAs based on one-way ANO​VA with Dunnett’s multiple comparisons test. **(D)** CCER CRI​SPRi HCT116 cells transduced with sgULK1 or sgATL2 were starved, harvested, and Western blotted. **(E)** Quantification of data from D. Data presented as mean ± SD of three biological replicates. P value indicates one-way ANO​VA with Dunnett’s multiple comparisons test (**, P < 0.005). **(F)** CCER CRI​SPRi HCT116 cells stably transduced with the indicated sgRNAs were starved and immunostained with LAMP2 antibody. Scale bar represents 20 µm. Inset represents 3× enlargement of boxed area. **(G)** Quantification of data from F. An average of 100 cells per condition were quantified from three biological replicates. P value indicates one-way ANO​VA with Dunnett’s multiple comparisons test (**, P < 0.01; ***, P < 0.001). **(H)** mCherry-eGFP-LC3B CRI​SPRi HCT116 cells were starved for 8 h and measured by FACS similar to the EATR assay. Data presented as mean ± SD of three biological replicates. P value indicates one-way ANO​VA with Dunnett’s multiple comparisons test (***, P < 0.0005). **(I)** CCER CRI​SPRi HCT116 cells expressing sgATG10 or sgATL2 were starved for 8 h, fixed, and immunostained with LC3B antibody. Scale bar represents 20 µm. **(J)** HCT116 cells expressing sgATG10 or sgATL2 were starved for 16 h, harvested, and subjected to Western blot. **(K)** Quantification of data from J. Data presented as mean ± SD of n > 4 biological replicates. P value indicates unpaired, two-tailed student t test (*, P < 0.05; **, P < 0.005; ***, P < 0.001; ****, P < 0.0001). **(L)** EATR CRI​SPRi HCT116 cells expressing sgATL2 and the indicated ATL constructs were starved for flow cytometry. Data presented as mean ± SD of three biological replicates. P value indicates one-way ANO​VA with Dunnett’s multiple comparisons test (***, P < 0.005; ****, P < 0.0001). **(M)** CCER CRI​SPRi HCT116 cells expressing sgATL2 and the indicated overexpression constructs were treated the same as L, harvested, and subjected to Western blot.

Both the HTML and PDF versions of the article have been corrected. These errors appear only in print and PDF versions downloaded on or before October 18, 2018.

